# Management of ketogenic diet therapy in patients with ileostomy: A report of 2 cases

**DOI:** 10.1002/ncp.70164

**Published:** 2026-08-25

**Authors:** Braden J. Baker, Abigail E. Olbina, Marissa A. Godfrey, Annie E. Graber, Taylor N. Parrish, Sarah Isis R. Delima

**Affiliations:** ^1^ Indiana University School of Medicine Indianapolis Indiana USA; ^2^ Riley Hospital for Children at IU Health Indianapolis Indiana USA; ^3^ Department of Neurology Indiana University School of Medicine Indianapolis Indiana USA

**Keywords:** fluids–electrolytes/acid–base, nutrition assessment, pediatric medicine, pediatrics, rehabilitation

## Abstract

This case series explores the feasibility and clinical considerations of implementing the ketogenic diet therapy (KDT) for epilepsy management in patients with ileostomies. Two pediatric patients with medication‐resistant epilepsy and ileostomies continued KDT under clinical supervision. The first patient had an ileostomy placed while on KDT. Fluids and electrolytes were increased initially, although sodium (Na) levels remained within a normal range. The second patient had an ileostomy before starting KDT. Hyponatremia and acidosis occurred initially. With routine laboratory monitoring, and electrolyte and bicarbonate supplementation, normal laboratory values were achieved. Overall fluid needs did not change. Both patients maintained adequate hydration and electrolytes. Seizure control was not compromised in either case while having an ostomy. This study supports the feasibility of KDT in patients with ileostomies, while also highlighting the need for individualized monitoring of hydration status and electrolytes, specifically sodium levels.

## INTRODUCTION

Ketogenic diet therapy (KDT) is a high‐fat, adequate‐protein, and low‐carbohydrate diet that uses ketones as the primary fuel source to produce ketone bodies. KDT is indicated in patients with epilepsy who have failed two or more anti‐seizure medications (ASMs) and for specific metabolic conditions such as glucose transporter‐1 deficiency syndrome and pyruvate dehydrogenase deficiency.^1^ KDT is believed to work by altering neurotransmitter levels such as adenosine, norepinephrine, glutamate, and γ‐aminobutyric acid. KDT has proven successful by mechanisms such as influencing mitochondrial function and reactive oxygen species, decreasing inflammation, and increasing immune function.^2^

The most recent KDT consensus report from 2018 outlined suggested screening laboratory studies prior to implementation of KDT, including complete blood count, electrolytes, liver and kidney function tests, lipid profile, and metabolic profile such as total and free carnitine levels and acylcarnitine profile. Initiation of KDT is commonly done while admitted for observation; however, it can be started on an outpatient basis. Side effects of the diet can include constipation, delayed gastric emptying, increase in gas and bloating, emesis, hyperlipidemia, metabolic acidosis, and nutrient deficiencies. There are few studies discussing long‐term complications, but those noted include bone fractures, dyslipidemia, and kidney stones.^1^

Patients who cannot maintain adequate oral intake or nutrition to meet their metabolic demands are considered for feeding tube placement, guided by a nutrition protocol constructed with interdisciplinary members to address the patient's individualized dietary needs.^3^

Some further challenges can arise in patients with colonic growths, malignancies, inflammatory bowel diseases, or perforation peritonitis resulting in the need for a stoma to divert feces away from distal portions of the bowel. Patients can receive an ileostomy, which is created from their small bowel, or a colostomy, which stems from their colon.^4^ Ileostomies can either be temporary or permanent, depending on the clinical situation. Their purpose is to evacuate stool from the body via the ileum rather than through the usual route of the anus, allowing waste to bypass damaged areas of the intestine, reducing pain, inflammation, and discomfort.^5^ The prevalence of ileostomies for children varies depending on their medical conditions and specific requirements with one study showing that, out of 336 children who needed a stoma, 57% or 192/336 required ileostomy.^6^ The use of KDT and ileostomies together in children is not documented in medical literature.

The ileum serves to absorb most nutrients, including carbohydrates, fats, and water. Ileostomies can greatly impact the surface area of nutrient absorption. Patients with stomas require specific considerations regarding disturbances in water and electrolyte balance, and this can be influenced by the amount of output in the stoma bag. Since nutrient absorption can be impaired in patients with stomas, dietary modifications need to occur in order to replenish what the patient is losing.^7^ Because KDT relies heavily on fats, nutritional deficiencies can be further impacted in patients who require KDT and have an ileostomy. Ileostomy output can also further complicate a patient's fluid and water balance which can be exacerbated by KDT.

KDT is individualized and can be adjusted based on specific needs. Patients can receive different ketogenic ratios, which refers to the amount of fat compared to the amount of combined protein and carbohydrates. This ratio is often used for dosing purposes because KDT is used to decrease seizure burden. Depending on a patient's specific diet needs, homemade electrolyte water can be used. For example, instead of using store‐bought electrolyte rehydration drinks, individuals can create their own at home using solutions such as Morton Salt and baking soda to fulfill sodium, chloride, and potassium needs.

Despite the established use of KDT in epilepsy and the common use of ostomies in gastrointestinal care, there is no published literature of the two used in conjunction. Therefore, the management for electrolyte balances, water composition, and nutrition is not widely known. Here we discuss two cases, detailing the nutritional course and changes, and seizure and disease progression. Both of these patients had an ostomy as well as intractable epilepsy for which KDT was used as treatment.

### Case presentations

#### Patient 1

Patient 1 is a 16‐year‐old female with history of congenital cytomegalovirus, microcephaly, spastic quadriplegic cerebral palsy, generalized intractable epilepsy, and chronic dysmotility requiring nutrition via gastrojejunostomy (G/J) tube who was started on KDT for seizure control. Before KDT, patient 1 had several daily tonic seizures. After KDT, tonic seizures resolved, and she was weaned off of her ASMs. In November 2023, an ileostomy was performed due to chronic dysmotility and KDT was continued. All nutritional companies used can be seen in Table 1.

**Table 1:** Manufacturers of supplements/formulas included in this study.

Supplement/formula	Company	Location of company
KetoVie Peptide Plant Based 4:1	Ajinomoto Cambrooke Inc.	Ayer, MA
Liquigen®	Nutricia	White Plains, NY
Complete Amino Acid Mix	Nutricia	White Plains, NY
Flintstones Complete Multivitamin™	Bayer Consumer Health	Whippany, NJ
Kate Farms Peptide 1.5	Kate Farms®™	Goleta, CA
Morton® Salt Substitute	Morton® Salt, Inc.	Overland Park, KS
Morton® Lite salt	Morton® Salt, Inc.	Overland Park, KS
Magnesium Vitality	Natural Vitality®	Sunrise, FL
Visibiome™ Extra Strength Probiotic	Exegi Pharma	Rockville, MD
NanoVM® t/f	Solace Nutrition	North Stonington, CT
K‐Phos Neutral®	Beach Pharmaceuticals	Tampa, FL

The patient's feeding plan prior to her ileostomy was a 3.8:1 ketogenic ratio as follows:

Formula recipe:

– 1100 mL KetoVie Peptide 4:1 liquid
– 20 mL Liquigen
– ½ tsp baking soda
– ¾ tsp Morton Salt Substitute
– ½ tsp calcium carbonate powder
– 995 mL water
– Total volume: 2115 mL
– Run at 95 mL/h via J‐tube until finished

Additional supplements:

– K‐Phos Neutral 250 mg daily
– Ferrous sulfate 325 mg daily
– Flintstones Complete Multivitamin: ½ tablet daily
– Levocarnitine 100 mg/1 mL: 250 mg = 2.5 mL twice daily
– Zinc: 30 mg daily

This feeding plan provided:

– Na: 2417.5 mg (dietary reference intake [DRI]: 1500 mg) 161%
– K: 3415 mg (DRI: 2300 mg) 148%
– Cl: 3355 mg (DRI: 2300 mg) 146%
– Water: 1874 mL

After the ileostomy procedure, the patient was admitted multiple times over a few months due to feeding intolerance and decreased ostomy output. Despite an increase in fluid and other interventions, eventually an exploratory laparotomy was performed, which discovered a volvulus. Once the volvulus was corrected, her feeding intolerance resolved. Her nutrition course is seen in Table 2. The following dietary alterations were made based on the patient's electrolyte status:

**Table 2:** Patient 1 lab progression with diet alterations.

	Reference range	Start of diet	Week 5	Week 12	Week 13	Week 31	Week 40	Week 47	Week 52
Sodium (mmol/L)	135–145	142	141	141	137	143	142	143	142
Potassium (mmol/L)	3.5–5.5	5.6*	4.4	4.3	5.5	4.1	4.9	5.2	5.1
Chloride (mmol/L)	98–108	101	99	95**	104	103	101	106	103
CO_2_ (mmol/L)	22–29	25	26	33*	25	27	26	24	26
Anion Gap (mmol/L)	3–11	16*	16*	13*	8	13*	15*	13*	13*
Glucose (mg/dL)	70–99	75	79	87	68	100*	70	80	80
Beta‐OH Butyrate (mmol/L)	0.02–0.27	N/A	4.50*	N/A	N/A	2.7*	4.39*	4.47*	3.81*

* Laboratory level is higher than reference range.

** Laboratory level is lower than reference range.

Week 5: Total fluids were increased by adding 360 mL of homemade electrolyte water.

Week 6: Decreased ratio to 3.4:1 due to continued intolerance to feeds.

Week 12: Increased electrolyte water by 4 oz (total: 480 mL/day).

Week 14: Decreased ratio further to 3:1 (thought due to continued intolerance); on total parenteral nutrition (TPN) during continued intolerance in admission. Started TPN as a 3‐day bridge to restarting enteral nutrition after undergoing repair for small bowel volvulus around ileostomy. Slow feeds, also known as trickle feeds, were started at a very low rate: <5 mL/h. These feeds were started to prevent any intestinal atrophy, which can lead to long‐term malabsorption issues. After the trickle feeds, the patient was advanced to full KDT J‐tube feeds.

Week 30: Decreased water to 800 mL/day.

Week 45: Increased ratio to 3.35:1 (due to supposed increase in seizures per family report); decreased motility meds.

Week 47: Increased calories by 5%, decreased daily fluids by 120 mL/day of water and increased zinc to 30 mg in the morning and 15 mg in the evening(1.19 mg/kg/day). Zinc was increased due to labs showing low plasma concentrations.

Week 54: Increased iron supplement due to low ferritin levels.

After these changes to the patient's nutrition plan, her final diet had a 3.4:1 ketogenic ratio as follows:

Formula recipe:

– 1225 mL KetoVie Peptide 4:1 liquid
– 7 g Complete Amino Acid Mix
– ½ tsp baking soda
– ¾ tsp Morton Salt Substitute
– ½ tsp calcium carbonate powder
– 680 mL water
– 500 mL homemade water and electrolytes*
– Total volume: 2410 mL
– Run at 100 mL/h via J‐tube until finished

*Homemade water and electrolytes recipe:

– 960 mL water
– ½ tsp baking soda
– ½ tsp Morton Salt Substitute

Additional supplements:

– K‐Phos Neutral 250 mg daily
– Ferrous sulfate 325 mg daily
– Flintstones Complete Multivitamin: ½ tablet daily
– Levocarnitine 100 mg/mL: 250 mg = 2.5 mL twice daily
– Zinc: 30 mg in the morning daily, 15 mg in the evening daily

This feeding plan provided:

Water and electrolytes content:

– Na: 2901 mg (DRI: 1500 mg) 193%
– K: 4200 mg (DRI: 2300 mg) 183%
– Cl: 4099 mg (DRI: 2300 mg) 178%
– Water: 2148 mL

Since her final feeding plan was established, the patient has been stable and has not been readmitted. Following her family's report at week 45, video electroencephalography demonstrated no increase in seizure frequency, with seizures remaining well controlled with KDT.

#### Patient 2

Patient 2 was a 14‐year‐old female with a history of trisomy 18 (Edwards syndrome), pancreatitis, tracheostomy dependence, bicuspid aortic valve, abdominal compartment syndrome with ileostomy placement, and feeding via G/J tube, who is now deceased. This patient had an ileostomy 10 months prior to starting KDT, placed due to a long‐standing history of chronic colonic dysmotility.

Prior to KDT, the patient's feeding plan consisted of the following:

Formula recipe:

– 500 mL Kate Farms Peptide 1.5 (Adult)
– 580 mL water
– 1 tsp iodized salt
– ¾ tsp Morton Lite Salt
– Total volume: 1080 mL
– Give 2 daytime feeds of 290 mL each. At night for about 9.5 hours, run remaining formula (580 mL) at 60 mL/hr via J‐tube until finished

Additional fluids:

– 790 mL water daily

Additional supplements:

– Ferrous sulfate 325 mg: ½ tablet daily
– Vitamin C powder: 200 mg daily
– NanoVM t/f: one scoop daily
– Magnesium Vitality: ¾ tsp daily

This feeding plan provided:

– Na: 3769 mg (DRI: 1500 mg) 251%
– K: 2866 mg (DRI: 2300 mg) 125%
– Cl: 6793 mg (DRI: 2300 mg) 295%
– Water: 1825 mL

Once initiating KDT, a subtle, periodic transition was started from the Kate Farms Peptide 1.5 (Adult) to KetoVie Plant‐Based 4:1 liquid. During the initiation, formula recipe changes were made based on the patient's electrolytes, CO_2_ levels, and ketosis as determined by β‐hydroxybutyrate levels, as seen below. These changes can also be viewed in Table 3. After the patient had been on KDT for about 3 months, her mother reported improvement with overall seizure activity. Some other complications arose while on KDT, however. The patient displayed consistently low CO_2_ levels (<21 mmol/L) and suffered from intermittent intussusception requiring admission on week 13. This required switching the diet to nothing by mouth (NPO) status, disrupting the patient's electrolyte balance and requiring additional repletion. Acidosis occurred secondary to lack of electrolyte supplementation while on NPO status. Intussusception was potentially due to G/J tube dysfunction with low suspicion of connection to KDT.

**Table 3:** Patient 2 lab progression with diet alterations.

	Reference range	Start of diet	Week 3	Week 4	Week 5	Week 7	Week 10	Week 16
Sodium (mmol/L)	135–145	141	134**	133**	139	137	134**	139
Potassium (mmol/L)	3.5–5.5	4.1	4.3	4.6	4	4	4.3	3.5
Chloride (mmol/L)	98–108	112*	103	98	105	106	109*	112*
CO_2_ (mmol/L)	22–29	20**	21**	21**	20**	20**	18**	11**
Anion Gap (mmol/L)	3–11	9	10	14*	14*	11	7	18*
Glucose (mg/dL)	70–99	91	88	78	83	N/A	70	68
Beta‐OH Butyrate (mmol/L)	0.02–0.27	N/A	0.42*	1.89*	2.31*	N/A	2.75*	4.00*

* Laboratory level is higher than reference range.

** Laboratory level is lower than reference range.

Week 3: Increased baking soda to ½ tsp/day and salt to 1¼ tsp/day.

Week 4: Increased total volume by 30 mL.

Week 5: Increased baking soda to ¾ tsp/day.

Week 9: Increased baking soda to 1 tsp/day and increased calories.

Week 11: Increased baking soda to 1¼ + 1/8 tsp/day and increased calories.

Week 13: Switched to NPO status due to hospital admission.

Week 14: Resumed KDT.

Week 16: Increased baking soda to 2 tsp/day due to low inpatient levels of Na and CO_2._

The final diet with a 2.86:1 ketogenic ratio consisted of the following:

Formula recipe:

– 570 mL KetoVie Plant‐Based 4:1 liquid
– 10 g Complete Amino Acid Mix
– 500 mL water
– 2 tsp baking soda
– 1¼ tsp iodized salt
– ¾ tsp Morton Lite Salt
– Total volume: 1080 mL
– Give 2 daytime feeds of 290 mL each. At night for about 9.5 hours, run remaining formula (580 mL) at 60 mL/hr via J‐tube until finished

Additional fluids:

– 870 mL water

Additional supplements:

– Ferrous sulfate 325 mg: ½ tablet daily
– Vitamin C powder: 200 mg daily
– NanoVM t/f: one scoop daily
– Magnesium Vitality: ½ tsp daily
– Visibiome Extra Strength Probiotic: one to two packets daily

This feeding plan provided:

– Na: 6965 mg (DRI: 1500 mg) 464%
– K: 2319 mg (DRI: 2300 mg) 101%
– Cl: 7702.5 mg (DRI: 2300 mg) 335%
– Water: 1820 mL

The patient discontinued KDT on week 17 due to parent preference after acute illness requiring extended hospitalization.

## DISCUSSION

As epilepsy is a debilitating neurological disorder, significant efforts have been dedicated to improving its management while minimizing treatment‐related side effects. The primary goals of treatment include identifying effective ASMs or therapies for individual patients, minimizing treatment‐related side effects, and ensuring sustained long‐term seizure control.^1^, ^2^ Despite advances in ASMs, approximately 30%–40% of patients continue to have medication‐resistant epilepsy, which has led to increased use of alternative therapies such as KDT.^2^

KDT is a well‐established treatment option for patients with medication‐resistant epilepsy, particularly those who have failed two or more appropriately chosen ASMs or who have epilepsy syndromes known to respond well to dietary therapy.^1^, ^2^ In these select patients, KDT commonly results in meaningful seizure reduction, with approximately 50%–60% achieving at least a 50% reduction in seizures and 10%–15% achieving seizure freedom.^2^, ^8^ While higher response rates have been reported in select cohorts, these outcomes are not representative of most patients. Nonetheless, for many individuals, KDT can be a life‐changing intervention.

Although complications such as hypoglycemia, metabolic acidosis, dehydration, and electrolyte abnormalities can occur, these risks are reduced when KDT is initiated gradually. Gradual initiation typically involves slowly advancing the ketogenic ratio over several days, avoiding prolonged fasting, and closely monitoring serum glucose, electrolytes, and acid–base status during initiation.^9^ Compared with ASMs, KDT has a different adverse‐effect profile which includes die metabolic complications requiring close monitoring.^1^, ^2^

Dehydration is a particularly important consideration with KDT, as low‐carbohydrate diets promote increased urinary sodium and water losses through a diuretic and natriuretic effect, especially during early phases of ketosis.^2^, ^8^ Electrolyte abnormalities, including hyponatremia, are therefore more likely to occur during diet initiation and require proactive management.

In patients with ileostomies, the risk of dehydration and electrolyte imbalance is further increased. These patients lack colonic reabsorption of water and sodium, resulting in higher baseline fluid and sodium losses.^3^, ^10^ While fluid losses occur in patients with ileostomies regardless of diet, the addition of KDT may exacerbate these losses due to its diuretic effects. In the two patients discussed, hydration status and sodium balance required close monitoring, and both patients required sodium supplementation. This suggests a shared physiologic response rather than isolated events. Although the small sample size limits broader conclusions, these cases highlight the importance of careful fluid and electrolyte management when implementing KDT in patients with altered gastrointestinal anatomy.

## CONCLUSION

This case series describes the implementation of KDT in two patients with ileostomies and highlights the importance of close monitoring during diet initiation and maintenance. In both patients, careful attention to hydration status, serum electrolytes, and metabolic parameters was required. Laboratory monitoring after each ratio or recipe change, followed by regular outpatient follow‐up, allowed for early identification and management of dehydration and electrolyte abnormalities.

While these cases demonstrate that KDT can be successfully implemented in select patients with ileostomies, there are several study limitations that need to be addressed. These findings are limited to the two patients described and should not be generalized to all individuals with epilepsy and ostomies. Patients with altered gastrointestinal anatomy may be at increased risk for complications during KDT and require individualized monitoring and multidisciplinary support. Further studies are needed to better define optimal monitoring strategies and long‐term outcomes in this higher‐risk population.

For this case report, there is no comparison group and a cause‐and‐effect relationship cannot be determined. The two cases differ substantially in both underlying conditions, complications, and timeframe of starting the ketogenic diet in relation to ostomy placement. This introduces heterogeneity and prevents standardization of treatment. Lastly, this study relies on retrospective data, introducing the potential for information bias including incomplete or inaccurate reporting, variable laboratory timing, and subjective caregiver reporting of seizure activity.

## AUTHOR CONTRIBUTIONS

Taylor N. Parrish and Sarah Isis R. Delima developed the study concept and design. Braden J. Baker and Abigail E. Olbina collected data from patient cases. Braden J. Baker, Abigail E. Olbina, Marissa A. Godfrey, and Annie E. Graber drafted the manuscript. Taylor N. Parrish contributed to the patient cases and provided critical feedback to the manuscript. Sarah Isis R. Delima supervised the project and led group meetings. All authors critically revised the manuscript, approved the final version for publication, and agreed to be accountable for all aspects of the work.

## CONFLICT OF INTEREST STATEMENT

None declared.

## ETHICS STATEMENT

This study with IRB #25534 was reviewed by the IU Human Research Protection Program (HRPP) and determined to be exempt from full review as a retrospective case series. Informed consent was obtained from the guardians of the patients.
